# Diagnostic and therapeutic update of mantle cell lymphoma (MCL): analysis of seven cases treated in a centre in one year

**DOI:** 10.3332/ecancer.2016.627

**Published:** 2016-03-17

**Authors:** Carmen Herrero-Vicent, Isidro Machado, Carmen Illueca, Amparo Avaria, Claudia Salazar, Abraham Hernandez, Sergio Sandiego, Javier Lavernia

**Affiliations:** 1 Medical Oncology Department, Instituto Valenciano de Oncología, Valencia, Spain; 2 Pathological Anatomy Department, Instituto Valenciano de Oncología, Valencia, Spain; 3 Haematology Unit, Instituto Valenciano de Oncología, Valencia, Spain

**Keywords:** mantle cell lymphoma, prognostic factors, chemoimmunotherapy, new drugs

## Abstract

Mantle cell lymphoma (MCL) is an infrequent subtype of non-Hodgkin’s lymphoma (NHL) and represents between 4–8% of adult lymphomas. Recently an increase in its incidence to 1–2 cases/100,000 inhabitants/year has been observed. The first line of treatment is based on chemoimmunotherapy and depends on age and the initial stage at diagnosis. There are no second line or successive treatments. There are currently several drugs available that provide acceptable results.

## Introduction

MCL accounts for 4–8% of all adult non-Hodgkin’s lymphomas (NHL). Recently an increase in its incidence to one to two cases/100,000 inhabitants/year has been observed [1], making it possible for it to be catalogued as a rare tumour as the Surveillance of Rare Cancers in Europe (RARECARE), which considers those with an incidence lower than six cases/100,000 inhabitants/year to be rare [1]. It is a specific clinicopathologic subtype of NHL that displays an aggressive clinical behaviour. It is considered incurable, with median survival rates between three and five years. It is characterised by translocation t(11;14) and immunohistochemical overexpression of cyclin D1 [1].

Just as we have prognostic indices for follicular lymphoma and diffuse large cell lymphoma like FLIPI and IPI respectively, since 2008 there has been an international prognosis index for MCL, mantle cell lymphoma internal prognostic index (MIPI) that includes age, Eastern Cooperative Oncology Group (ECOG), lactate dehydrogenase (LDH), and leukocyte count establishing high-, intermediate-, and low-risk groups [2] (Tables 1 and 2).

The therapeutic approach is based on the combination of immunotherapy and chemotherapy followed either or not by the transplant of haematopoietic stem cells. With regard to relapse, there is no standard treatment, but with the development of new drugs, there is the choice of rituximab with or without temsirolimus, ibrutinib, bortezomib, or lenalidomide [3].

## Materials and methods

In the present work, we offer an updated review of the current diagnostic criteria and therapeutic options available, as well as of the new drugs that can be incorporated in the treatment of relapsed MCL in the near future. In this present review we have analysed the clinical, histological, immunohistochemical, and molecular characteristics of seven MCL patients diagnosed in our centre between February 2014 and February 2015 and the treatment administered.

Clinicopathological and therapeutic data were collected from the clinical history. The histologic subtype and the immunohistochemical profile were taken from the anatomopathological report in search of the following immunomarkers: CD5, CD23, CD43, cyclin D1, BCL 2, and BCL 6.

The patients were diagnosed and staged according to the modified Ann Arbor classification (Costworlds 1989) after carrying out an analytical control with haemogram, complete biochemistry, including LDH, cervico-thoraco-abdomino-pelvic computerised axial tomography (CT), ganglionary biopsy, or biopsy of the accessible affected extranodal tissue, and bone marrow biopsy.

## Results

Seven patients were diagnosed with MCL and treated in the Instituto Valenciano de Oncología (IVO) Medical Oncology or Haematology Department. The average age of our patients was 63.5 years (ranging from 50–73 years) with a male to female ratio of 6:1 (Table 3). Five of the seven patients displayed advanced stages at diagnosis: one stage III case and the four remaining cases in stage IV with bone marrow infiltration in all of them. At the time of diagnosis, only one of the seven patients displayed B symptoms (Table 3).

**Figure 1.**
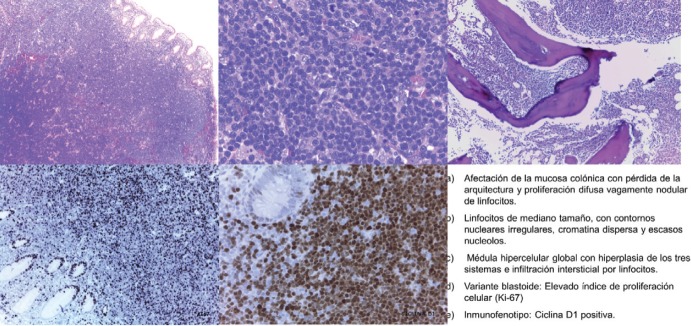
Pathological anatomy of the patient diagnosed after biopsy of a colonic polyp. a) Involvement of the colonic mucosa with loss of structure and vaguely diffuse nodular lymphocyte proliferation. b) Medium sized lymphocytes with irregular nuclear outlines, disperse chromatin, and scant nucleoles. c) Global hypercellular medulla with hyperplasia of the three systems and interstitial infiltration by lymphocytes. d) Blastoid variant: elevated index of cellular proliferation (Ki-67). e) Immunophenotype: cyclin D1 positive.

Case 5, a male patient of 73 years of age, who was evaluated by Haematology in November 2012 for asymptomatic neutropaenia without apparent cause (N 820 mcg/L). The study was carried out by means of immunophenotyping peripheral blood cells with results compatible with B-chronic lymphoproliferative disorder without the criteria for chronic lymphocytic leukaemia. An extended CAT scan revealed supra and infraclavicular adenopathies. As the adenopathies were not accessible for biopsy, a bone marrow aspiration biopsy was performed that revealed low-grade B-cell lymphoproliferative disorder. The tumour committee decided in favour of monitoring as treatment criteria were not present. The patient was sent to the Digestive Department in December 2013 because of a positive fecal occult blood test in colorrectal cancer screening (CCR). A colonoscopy was performed that focused on nine submucosal polyps throughout the colon. The anatomopathological study of the entirety of the extirpated polyps revealed blastoid variant MCL with a pattern of lymphomatoid polyposis. An extended study was requested. Computerised tomography (CT) showed stability of the supra and infradiaphragmatic adenopathies with respect to the October 2013 CT. PET/CT were within the normal range. Analysis for LDH and beta2 microglobulin were within range. Bone marrow aspiration/biopsy revealed global hypercellular marrow with hyperplasia of the three systems with interstitial, paratrabecular, and nodular infiltration by NHL. CD20+++ and cyclin D1++ were compatible with infiltration by MCL. Cytology showed increase in lymphocytes suggestive of marrow infiltration by NHL (Figure 1). The molecular biology study showed translocation t(11;14) and BCL1-IgH gene fusion for positive MCL diagnosis.

Histologically, three patients displayed a blastoid variant whereas the remaining four displayed a classic variant. In five of the cases, translocation t(11;14) could be determined and verified, while in the other two cases it was not analysed. However, the seven patients were positive for cyclin D1, CD5, and CD43, and BCL2, and negative for BCL6 (Table 4).

Six of the cases received rituximab-CHOP as front-line treatment, while the youngest patient received rituximab-Hyper-CVAD. One case received local radiotherapy upon presenting stage II at the end of chemotherapy. Following systemic treatment, CT showed a complete response for six patients; targeted PET for three, and in the case of the patient with intestinal condition, study was completed with a colonoscopy. In all the cases with diagnosed bone marrow infiltration that presented a complete radiological response following systemic treatment, a bone marrow biopsy confirmed the absence of infiltration by neoplastic cells.

Six of the cases were included in the MIPI high-risk subgroup, and the remaining case in the low-risk group (Table 3).

With an average monitoring time of 10.8 months, one of the patients, diagnosed with a blastoid variant MCL (case number 7), had a ganglionary relapse and was being treated with rituximab-bendamustine as second-line. The remaining six cases remain disease-free.

## Discussion

The median age at diagnosis is found to be 68 +/-8 years. In 75% of the cases, MCL affects men and is more frequent among Caucasians [3]. Also, in our case history the average age was 63.5 years (range 50–73 years), all male except one.

Clinically the symptoms collected in the literature are nonspecific, although usually it appears as a constitutional disorder and B symptoms, which is why it is important to make a complete anamnesis at the time of staging [3]. Other forms of presentation can be the appearance of multiple peripheral adenopathies, hepatomegaly/splenomegaly, and less frequently a secondary intestinal obstruction to intestinal lymphomatoid polyposis [4]. In our series, one patient presented with B symptoms, five presented clinically as nonspecific, and in one case diagnosis was coincidental.

In order to make the diagnosis, an exeresis/biopsy of an accessible adenopathy and then evaluation by means of morphologic study with a haematoxylin and eosin stain, nuclear immunohistochemical expression for cyclin D1, and confirmation via molecular studies using FISH, cytogenetics, or determination of the specific gene fusion after translocation t(11:14) is recommended [1].

An analytical control should be included in the extension study to determine LDH, serology for the hepatitis B virus, human immunodeficiency virus, cervico-thoraco-abdomino-pelvic CT, and bone marrow biopsy [1]. Although bone marrow infiltration can meet diagnostic criteria, ganglionary biopsy is always recommended [1]. There is no consensus on the use of PET/CAT as part of the initial study, but it could be recommended in stages I/II before local radiotherapy [1]. Under the Lugano classification, PET/CT has become essential for basal staging to increase the precision of the response to treatment. In MCL, however, it presents as a low specificity and sensitivity for identifying intestinal effect, which is why it does not replace carrying out other diagnostic explorations [5]. The study of different locations is justified according to the case history: in patients with gastrointestinal symptoms or Waldeyer’s ring involvement, a gastroscopy and colonoscopy should be performed [1]. For patients with blastoid variant mantle lymphoma, elevated LDH, and/or neurological history, lumbar puncture is indicated [1]. A left ventricular ejection fraction should be performed in patients who receive treatment with anthracyclines [1].

Several works describe the disease in stages III and IV [4] with the presence of multiple adenopathies in 90% of the cases, although one in four cases has extranodal involvement in its presentation for e.g. bone marrow (75–80%), spleen (55%), liver (35%), gastrointestinal tract (20%), and Waldeyer’s ring (10%) [4]. The involvement of the central nervous system is rare, but in the course of the disease it can appear in up to 10% of patients [4]. In our series, five of the seven cases presented with advanced disease (one stage III case and four stage IV cases). On diagnosis, patient number 5 presented disseminated disease with digestive involvement, an infrequent form of presentation of MCL that was diagnosed by means of biopsy of the polyps found in the colonoscopy as a result of a fecal occult blood test. However, it is difficult to determine whether this is primary gastric (extranodal) mantle cell lymphoma with a lymphomatoid polyposis pattern or a secondary infiltration of the intestinal mucosa by a nodal mantle cell lymphoma.

From the anatomopathological point of view, MCL is a neoplasm of small B-cell phenotype monomorphic cells with an irregular nucleus that originates in the mantle zone of the lymphoid follicle. There are four patterns of nodal involvement with partial or total loss of architecture, showing a diffuse, vaguely nodular proliferation with a mantle zone pattern or one similar to the follicular growth pattern [4].

Differential diagnosis should be made using the reactive processes (mantle zone hyperplasia), nodular lymphocyte predominant Hodgkin’s lymphoma, nodal marginal zone lymphoma, small lymphocytic lymphoma/chronic lymphocytic leukaemia, follicular lymphoma, lymphoblastic lymphoma (with blastoid variant MCL), and diffuse large B-cell lymphoma (with pleomorphic variant MCL) (Table 5).

The lymphocyte proliferation in MCL is monomorphic, with the absence of cells like centroblasts, immunoblasts, and paraimmunoblasts being very characteristic, as well as proliferation of pseudocentres that differentiate mantle lymphoma from other low-grade B-cell neoplasms. Additionally, different cellular variants exist. One being small cell variant which is similar to chronic lymphocytic leukaemia. The other that simulates marginal zone lymphocytes with clear cytoplasm or monocytoid B-cells, which are reminiscent of the proliferation centres of small lymphocytic lymphoma. Then the aggressive variants of higher cytological grade correspond to the pleomorphic variant (with cells similar to diffuse large B-cell non-Hodgkin’s lymphoma) and the blastoid with lymphocytes similar to the lymphoblasts of lymphoblastic lymphoma, with a high index of cellular proliferation (Ki-67), that entails a worse prognosis. The classic cytological variant constitutes 87%, small cell 3.6%, pleomorphic cell 5.9%, and blastoid variant 2.6% of the cases. Recent studies have demonstrated that miRNAs are considered important regulators of cellular behaviour. The increase in the expression of miR-15b likely plays an important role in the transformation of the classic variant of MCL to the aggressive variants [6]. In our series, four patients were diagnosed with the classic variant and three presented with a blastoid variant.

From the immunophenotype perspective, MCL expresses B-lymphocyte antigens such as CD19+, CD20+, CD22+, CD79+, PAX5, and IgM/IgD with lambda + light chain restriction. The most characteristic immunophenotypic markers are CD5+, FMC7+, CD45+, and intranuclear cyclin D1+ [7]. The expression of cyclin D1 is the most important characteristic with regard to immunophenotyping; in fact, in our series, all the patients displayed an overexpression of cyclin D1, but one must not forget that it can also occasionally be positive in hairy cell leukaemias, in 16% of myelomas, and very occasionally in some large B-cell lymphomas. It is worth stressing as well at this point that there are mantle cell lymphomas with classic morphology that lack t(11;14) and expression of cyclin D1.

In order to help to identify MCL with negative cyclin D1, the neural transcription factor (SOX11) has been proposed. Recent studies suggest SOX11 can play a role in the process of MCL cell differentiation and define two different subtypes of MCL through BCL6 transcriptional regulation [8]. However, SOX11 is not exclusive to MCL; it has also been identified in one-third of Burkitt’s lymphomas and in some cases of lymphoma /T-cell lymphoblastic leukaemia (Table 5). It also expresses negativity for the markers CD10-, CD23-, BCL6-, although there are aberrant phenotypes above all in the expression of CD10 and BCL6, and negativity for CD5, especially in the pleomorphic and blastoid variants (Table 5).

The overexpression of cyclin D1 or the detection of translocation t(11;14) are key diagnostic elements. In our series, they were detected in five patients. The origin of the immunophenotype expression of cyclin D1 is the characteristic translocation of MCL, t(11;14)(q13;q32) that takes place between the IGH gene and the cyclin D1-BCL1 (CCND1) gene, which is considered the primary genetic anomaly of MCL, documented in 70% of MCL cases [9]. An infrequent variant of MCL does not overexpress cyclin D1. This variant overexpresses cyclin D2 or D3 and expresses the transcription factor SOX11, maintaining the classic immunophenotype of mantle lymphoma.

With regard to treatment, it is important to differentiate between patients with localised disease (stages I–II) and advanced disease (III–IV):

Localised disease (stages I and II) accounts for less than 20% of patients. Front-line treatment must be based on immunochemotherapy [1].

- R-CHOP (three to four cycles) followed by external involved-field radiotherapy
- External involved-field radiotherapy exclusively for stage I [10]

Advanced disease (stages III–IV) accounts for the remaining 80% of patients. The objective is to obtain a complete remission, given that it is a key prognosis factor. But again on indolent variants, there is no standard plan, rather treatment depends on the age of the patient, their general state, and associated comorbidities.

There is an ample consensus on the treatment of the advanced disease, and groups with ample experience recommend [1] administering treatment with induction chemotherapy followed high-dose chemotherapy and autologous bone marrow transplantation (ABMT) to patients <65 years, without comorbidities, as it seems to improve the progression-free survival (PFS) and overall survival (OS) (Table 6). The three options we have are the following:

- Rituximab-CHOP x six cycles, ABMT if it responds [11].
- Nordic MCL2 protocol: This consists of the administration of three cycles of rituximab-CHOP alternating with three cycles of high doses of Ara-C, followed by ABMT with BEAM or BEAC [12].
- Rituximab-Hyper-CVAD x four cycles: In young patients this seems to improve the PFS to 4.8 years and OS 6.8 years, although it also increases toxicity significantly. It uses rituximab along with hyperfractionated cyclophosphamide, vincristine, doxorubicin, and dexamethasone, andit alternates this with high doses of methotrexate and cytarabine. If there is response after four cycles, it is completed with six cycles, and then with subsequent observation. In the absence of complete response, the patients will have to undergo high-dose chemotherapy and ABMT [13].

Intensive chemoimmunotherapy appears to improve survival (median PFS of 4.8 years and OS of 6.8 years) but with an associated mortality of 3–4%. It is commonly used in young patients, without comorbidities, with blastic variant, or a high-risk MIPI score. The subsequent administering of rituximab in maintenance has not shown any clear benefit in this group of young patients without comorbidities.

The first-line therapeutic recommendation in patients <65 years with comorbidities, unfit for chemotherapy at high doses and TAMO, or patients >65 years are:

- R-CHOP x six cycles, followed by rituximab (R) maintenance [14]
- R-CHOP x six cycles, followed by Rituximab (R) maintenance [14]
- R-CHOP x six cycles, followed by Rituximab (R) maintenance [14]

The administration of rituximab maintenance (every two months for two years) seems to increase the isolated limb perfusion (ILP) in the group of elderly patients or patients with comorbidities that contraindicate more aggressive treatments [15].

## Indolent variant

There is a variant of mantle cell lymphoma characterised by the presence of splenomegaly and bone marrow involvement. At the histological level they have a diffuse pattern, being positive for cyclin D1, but with a low rate of Ki67 proliferation. Watchful waiting may be considered if the patient is asymptomatic and if the following conditions are met; nonpleomorphic norblastoid variant, maximum tumour diameter less than 3 cm, Ki67 <30%, and normal levels of LDH and B2 microglobulin [16].

Most patients will require treatment at diagnosis, in our series all patients received first-line treatment at diagnosis, but the schema varied depending on the age of the patient, their general condition, and associated comorbidities. In first-line treatment there must always be an attempt to associate rituximab with the course of chemotherapy chosen, as observed with the cases we analysed. The majority of our patients received R-CHOP, except in the case of a young patient who received R-Hyper-CVAD.

## Relapse

There is no standard course, and the treatment also depends on the age of the patient, of the patient’s general condition, and on associated comorbidities. Drugs like bendamustine, temsirolimus, ibrutinib, and lenalidomide have proved useful (Table 7). The administration of a different course of treatment to that employed in first line or Antidian agents is recommended.

### R-bendamustine

In a phase III trial in which R-bendamustine was compared against R-fludarabine, a response rate of 83% versus 50% and a PFS of 30 months versus 11 months respectively, was demonstrated [17], for which reason it is considered that the combination rituximab-bendamustine is a good option in relapse.

In our series the only patient who presented a relapse was case number 5. He received R-bendamustine as a second-line course.

### Temsirolimus

In a phase III trial with 54 patients refractory or in relapse received temsirolimus 175 mg/week for three weeks, followed by 75 mg/week or 25 mg/week versus the treatment chosen by the investigator (gemcitabine or fludarabine). Significant differences were found in the rate of response: 22% for dose of temsirolimus of 75 mg, 6% for 25 mg, and 2% for the alternative treatment with gemcitabine or fludarabine, and a median PFS of 3.4 months for doses of 75 mg, 4.8 months for the dose of 25 mg, and 1.9 months for the alternate treatment [18], which has demonstrated the effectiveness of the mTor inhibitor. The combination of temsirolimus + rituximab has also demonstrated efficacy, given that in a phase II trial with 75 refractory or relapsed patients receiving temsirolimus 25 mg/week with rituximab, a response rate of 59% was demonstrated (19% complete responses) and a median PFS of 9.7 months was noted [19].

### Ibrutinib

In a phase II trial with 111 patients refractory or in relapse (with an average of three previous lines) who received ibrutinib 560mg/day oral continuum, a response rate of 68% (21% complete responses (CR)), and a median PFS of 13.9 months with an OS rate of 58% at 18 months were documented [20]. Ibrutinib can represent a hopeful alternative in patients with advanced disease in relapse with excellent tolerance and few side effects [21, 22].

### Bortezomib

In a phase II trial in refractory or relapsed patients receiving bortezomib in doses of 1.3 mg/m^2^, /days 1, 4, 8, and 11 of 21 days, a response rate of 40% and a median PFS of ten months are documented. It should be used with caution because it increases the risk of neurotoxicity G3-G4 in 13% [23].

### Lenalidomide

In a phase II trial with patients in third relapse and pretreated with bortezomib, receiving 25 mg/day x 21 days/28 days, response rates of 26% and a median PFS of 16 months were documented [24].

There are other agents currently in development; cyclin 4/6 (CDK 4/6) inhibitors as palbociclib or abemaciclib, phosphoinositol 3 kinase (PI3K) inhibitors as idelalisib, BCL2 inhibitors as venetoclax, histone deacetylase (HDACS) inhibitors as vorinostat. However, we do not have specific biomarkers that reveal in each patient what tracks and genetic alterations are involved [16, 25].

## Prognosis

Patients with MCL present globally a median OS of 36 months that drops to 18 months for the blastoid variant [26]. This proves that they are not cured, and are dying of their disease.

The main adverse anatomopathological prognostic factor is the tumour’s rate of mitosis (>50 /mm^2^) as well as a proliferation rate with Ki67 >40% [27].

In a retrospective study where we analysed various biological markers as prognostic factors, it was noted that the monocyte count >0.375 × 10(9)/L and elevated levels of LDH in the diagnosis were the two factors that were associated with a worse prognosis [28]. Other factors in poor prognosis are the blastoid variant, followed by the pleomorphic, trisomy 12, mutation of p53, and complex karyotypes related to an aggressive course.

At a clinical level, the forms associated with an improved prognosis are those that, even having involvement of the bone marrow and spleen, do not have lymph node involvement. Within the Ann Arbor classification, stages I, II constitute a group with good prognosis and more prolonged survival rates.

The MIPI demonstrates clearly the direct relationship existing between the index score prognosis and survival, so that the higher the MIPI score, the worse the survival rate. The MIPI catalogs patients in three categories according to their score [2] (Table 1 and 2).

- Low risk (44%), with median of OS 60% at five years
- Intermediate risk (35%), median OS of 51 months
- High risk (21%), with median of 29 months.

In our series, we calculated the MIPI and noted except for one case all others are included in the subgroup of patients at high risk. In the literature, 21% of mantle lymphoma is included in the high-risk subgroup, with a median survival of 29 months. A recently published study shows that gain or amplification of the MYC oncogene is associated with a greater tumour size, a greater percentage of intermediate or high risk according to the MIPI scale considered as an independent factor of poor prognosis [29].

## Conclusion

MCL constitutes 4–8% of adult NHL, and is characterised by the overexpression of cyclin D1 or the detection of the t(11;14). Its course is moderately aggressive and variable, so it is important to take into account the prognostic variables as the specific MIPI prognostic index.

Most of the patients require treatment at diagnosis. Treatment depends on the age of the patient, his general condition, and the associated comorbidities. For most patients with comorbidities, chemoimmunotherapy of induction type R-CHOP, R-CVP, or R-bendamustine, are good choices of treatment followed by maintenance rituximab for two years.

In those young patients and those under 65 years of age without associated comorbidities, more aggressive chemotherapy courses may be used after being evaluated for their response to induction treatment with chemotherapy to high doses and TAMO. In those patients who are not candidates for autologous transplant with response to induction scheme should be evaluated for maintenance treatment with rituximab.

There is no standard treatment for relapse, therefore it depends on the age of the patient and their general condition. The drugs to consider in cases of relapse, with or without rituximab, are: bortezomib, lenalidomide, bendamustine, and ibrutinib.

There are many studies with new drugs for MCL. For patients in relapse there is no standard treatment, hence further studies are needed to clarify as well as to reveal what would be the optimal sequence of treatment.

## Conflict of interest

The authors declare that they have no conflict of interest.

## Figures and Tables

**Table 1.** MIDI prognostic index score for mantle cell lymphoma.

POINTS	AGE (years)	ECOG	LDH /LSN (upper limit)	Leukocytosis (x 10^9^/L)
0	<50	0/1	<0.67	<6700
1	50–59	–	0.67–0.99	6700–9999
2	60–69	1/2	1–1.49	10000–14999
3	>70	–	>1–5	>15000

**Table 2.** MIDI prognostic index for mantle cell lymphoma.

RISK	POINTS	SURVIVAL AT FIVE YEARS
Low	0–3	60% alive at five years
Medium	4–5	51 months
High	>5	29 months

**Table 3.** Clinical and analytical characteristics of patients.

CASES	AGE	VARIANT	STATE	M.O.	LDH	LEUKOCYTOSIS	MIDI
CASE 1	66	Classic	IVA	SI	157	7700	6 HIGH
CASE 2	67	Blastoid	IVB	SI	522	14,000	7 HIGH
CASE 3	50	Classic	IIA	NO	150	5000	3 LOW
CASE 4	67	Blastoid	IIIA	NO	287	16,000	7 HIGH
CASE 5	73	Blastoid	IVA	SI	156	17,000	7 HIGH
CASE 6	50	Classic	IVA	SI	253	44,000	6 HIGH
CASE 7	72	Classic	IIA	NO	229	10,000	7 HIGH

**Table 4.** Anatomopathological and molecular characteristics of patients.

CASES	CYCLINE D1	CD5	CD43	BCL2	BCL6	CD23	REORDERING t(11;14)	KI67%
CASE 1	+++	+	++	+++	-	+	Yes	25
CASE 2	+++	+	++	+++	-	+	No	85
CASE 3	+++	+	++	+++	-	++	Yes	10
CASE 4	+++	+++	++	+++	-	-	Yes	75
CASE 5	+++	++	++	+++	-	-	Yes	50
CASE 6	+++	++	++	+++	-	+	No	20
CASE 7	+++	++	++	+++	-	-	Yes	20

**Table 5.** Differential diagnosis of B-cell lymphocytic neoplasms using immunohistochemical markers.

	Mantle cell lymphoma	Chronic lymphocytic leukaemia/ lymphocytic leukaemia	Follicular lymphoma	Marginal zone lymphoma	Lymphoplasmacytic lymphoma
CD3	-	-	-	-	-
CD5	+	+	-	-	-
CD10	-	-	+	+/-	+/-
CD20	+	+	+	+	+
CD23	+	+	+/-	+/-	+/-
BCL1	-	-	-	-	-
BCL2	+	+	+	+	+
BCL6	-	-	+	-	-
MIB/KI67	+	+	+	+	+
LEF1	-	+	-	-	-
CD160	-	+	-	-	-
CD200	-	+	-	-	+
SOX11	+	-	-	-	-
HGAL	-	-	+	-	-
LMO2	-	-	+	-	-
Stathmin	+	-	+	-	-
GCET1	-	-	+	-	-
IRTA1	-	-	-	+	-
MNDA	+	+	-	+	+
MYD88	-	-	-	-	+

**Table 6.** Summary of front-line treatment plans for mantle cell lymphoma.

	TREATMENT PLAN	RESPONSE RATE (%)	OVERALL SURVIVAL (months)
R-CHOP (7)	Rituximab 375 mg/m^2^ intravenous (IV) D1 Cyclophosphamide 750 mg/m^2^ IV D1 Vincristine 1.4 mg/m^2^ IV D1 Doxorubicin 50 mg/m^2^ D1 Prednisone 100 mg/day p.o. D 1–5 every 21 days × 6–8 cycles	RR 80% CR 35%	At four years 60%
Nordic MCL-2 protocol (8)	Cycles 1, 3, and 5. Maxi-CHOP Cyclophosphamide 1200 mg/m^2^ IV D1 Doxorubicin 75 mg/m^2^ IV D1 Vincristine 2 mg IV D1 Prednisone 100 mg IV D1–5 Cycles 2, 4, and 6: High dose Ara-C: ≤60 years 3 g/m^2^ IV; >60 years 2 g/m^2^ IV Rituximab 375 mg/m^2^ IV D0 as cycles 4, 5, and 6 After cycle 6 an extra dose of rituximab 375 mg/m^2^/day is administered as an *in vivo* purge, followed by ABMT with BEAM as conditioning regimen	RR 97% CR 54%	MIPI low-to-medium risk: - At six years 70% - At ten years 70% MIPI high-risk - At ten years 23%
R-HYPERCVAD/MTX/ Ara-C (9)	Rituximab 375 mg/m2 IV D0 of each cycle cycles 1, 3, 5, and 7 Cyclophosphamide 300 mg/m^2^/12 hours IV D 1–3 Mesna 600 mg/m^2^/day CIVI D1–3, beginning 1 hour before cyclophosphamide until 12 h our after Vincristine 2 mg IV D4 and D11 Adriamycin 50 mg/m^2^ IV D 4 Dexamethasone 40 mg/m^2^ IV D 1–4, D 11–14 cycles 2, 4, 6, and 8 Methotrexate 1000 mg/m^2^ CIVI 24 h D1: 200 mg/m^2^ IV in 2 hour followed by 800 mg/m^2^ IV in 22 hour Leucovorin 50 mg IV 12 hour after completion of methotrexate, followed by 15 mg/m2/6 hour until reaching serum methotrexate level <1 M Ara-C 3 g/m^2^ IV in 2 hour/12 hour D2 and D3 (four doses) The following cycle begins whenever neutrophils > 1 × 109/l and platelets > 6.0000	RR 62% CR 33%	PFS at 2 years 78%
R-CVP	Rituximab 375 mg/m^2^ IV D1 Cyclophosphamide 750 mg/m^2^ IV D1 Vincristine 1.4 mg/m^2^ IV D1 Prednisone 40 mg/m^2^/day p.o. D1–5 every 21 days × 6–8 cycles	RR 80% CR 25%	At three years 89%
R-bendamustine	Rituximab 375 mg/m^2^ IV D1 Bendamustine 90 mg/m^2^ D1 and D2 every 28 days × 6–8 cycles	RR 90% CR 50%	At four years 55%

**Table 7.** Summary of second-line treatment plans for mantle cell lymphoma.

TRIAL	TYPE	RESPONSE RATE (%)	PFS (months)
R-bendamustine versus R-fludarabine	III	83% 50%	30 11
R-temsirolimus 75 mg	II	59%	9.7
Ibrutinib 560 mg	II	68%	13.9
Bortezomib 1.3 mg/m^2^ 1, 4, 8, 11 × 21 days/28 days	II	40%	10
Lenalidomide 25 mg/d × 21 days every 28 days	II	26%	16
