# Erratum: Diagnostic and therapeutic update of mantle cell lymphoma (MCL): analysis of seven cases treated in a centre in one year

**DOI:** 10.3332/ecancer.2016.637

**Published:** 2016-04-27

**Authors:** Carmen Herrero-Vicent, Isidro Machado, Carmen Illueca, Amparo Avaria, Claudia Salazar, Abraham Hernandez, Sergio Sandiego, Javier Lavernia

**Affiliations:** 1 Medical Oncology Department, Instituto Valenciano de Oncología, Valencia, Spain; 2 Pathological Anatomy Department, Instituto Valenciano de Oncología, Valencia, Spain; 3 Haematology Unit, Instituto Valenciano de Oncología, Valencia, Spain

Table 3 was rendered incorrectly, please see the corrected table below.

On page 7, the line “• R-CHOP x six cycles, followed by rituximab (R) maintenance [14]” was repeated three times, whereas it should have only appeared once.

In Table 4: “CYCLINE D1”, should be “Cyclin D1” and “REORDERING”, should be “TRANSLOCATION”.

On page 5, “However, it is difficult to determine whether this is primary gastric (extranodal) mantle cell lymphoma with a lymphomatoid polyposis pattern or a secondary infiltration of the intestinal mucosa by a nodal mantle cell lymphoma.” should be “However, it is difficult to determine whether this is primary intestinal (extranodal) mantle cell lymphoma with a lymphomatoid polyposis pattern or a secondary infiltration of the intestinal mucosa by a nodal mantle cell lymphoma.”

On Page 8, “norblastoid variant” should be “non-blastoid variant”.

## Figures and Tables

**Table 3.** Clinical and analytical characteristics of patients.

CASES	AGE	VARIANT	STAGE	M.O.+	LDH	LEUKOCYTOSIS	MIDI
CASE 1	66	Classic	IVA	YES	157	7700	6 HIGH
CASE 2	67	Blastoid	IVB	YES	522	14000	7 HIGH
CASE 3	50	Classic	IIA	NO	150	5000	3 LOW
CASE 4	67	Blastoid	IIIA	NO	287	16000	7 HIGH
CASE 5	73	Blastoid	IVA	YES	156	17000	7 HIGH
CASE 6	50	Classic	IVA	YES	253	44000	6 HIGH
CASE 7	72	Classic	IIA	NO	229	10000	7 HIGH

